# Oxygen and immunity to *Leishmania* infection

**DOI:** 10.1128/iai.00504-24

**Published:** 2025-06-13

**Authors:** Bridget A. Bowman, Fernanda O. Novais

**Affiliations:** 1 Department of Microbial Infection and Immunity, College of Medicine, The Ohio State University 683676 , Columbus, Ohio, USA; University of California Merced, Merced, California, USA

**Keywords:** hypoxia, leishmaniasis, immune responses, parasites, oxygen

## Abstract

Oxygen availability plays a fundamental role in shaping immune responses to infections. Leishmaniasis, a disease caused by protozoan parasites of the genus *Leishmania*, manifests in a spectrum of clinical outcomes, ranging from localized cutaneous lesions to life-threatening visceral infections. Like many infections and chronic diseases, *Leishmania*-infected tissues are characterized by hypoxia. Despite the recognized importance of oxygen in immune regulation, our understanding of how hypoxia shapes the immune landscape in leishmaniasis remains in its early stages. Collectively, the published studies in leishmaniasis highlight the critical role of oxygen availability and hypoxia-inducible factor (HIF) in orchestrating immune responses, particularly within myeloid cells. Here, we review the literature on how oxygen availability and HIF signaling influence the immune response in leishmaniasis. By consolidating existing findings and identifying gaps in knowledge, we aim to inspire further research into the interplay between oxygen availability, immune function, disease progression, and therapeutic potential in leishmaniasis.

## HYPOXIA

Oxygen is essential for the survival of most eukaryotic organisms, serving as the foundation of energy production in cells (1). Through the process of oxidative phosphorylation, eukaryotic cells efficiently generate ATP, the primary energy currency essential for cellular function. However, when the supply of oxygen fails to meet the demands of tissues or cells, a state of hypoxia develops. This physiological imbalance is not uncommon and can arise under diverse conditions. Hypoxia plays a critical role in both physiological processes, such as adaptation to high altitudes and wound healing, and pathological states, including ischemia, tumor progression, and chronic infections (2). Understanding the mechanisms and consequences of hypoxia is vital for unraveling its impact on health and disease.

The understanding of how hypoxia can alter cell metabolism began in the early 20th century with Otto Warburg’s discovery of unique metabolic adaptations in tumor cells. He observed that these cells favor aerobic glycolysis despite its lower efficiency in ATP production compared to oxidative phosphorylation, a process now known as the “Warburg Effect.” This shift is critical for cellular adaptation to low oxygen environments, offering tumor cells a survival advantage (3). Decades later, the mechanisms by which cells sense and respond to hypoxia were discovered through the pioneering work of Drs. William Kaelin, Peter Ratcliffe, and Gregg Semenza in the early 1990s. Semenza identified the transcription factor hypoxia-inducible factor (HIF) as the central regulator of cellular responses to low oxygen levels, while Ratcliffe and Kaelin delineated the molecular pathways that govern HIF stability and activity. Their collective discoveries provided a detailed understanding of oxygen-sensing machinery at the cellular level (4–8). This transformative work earned the three scientists the Nobel Prize in Physiology or Medicine in 2019, solidifying their contributions as milestones in the field of hypoxia research.

**Fig 1:**
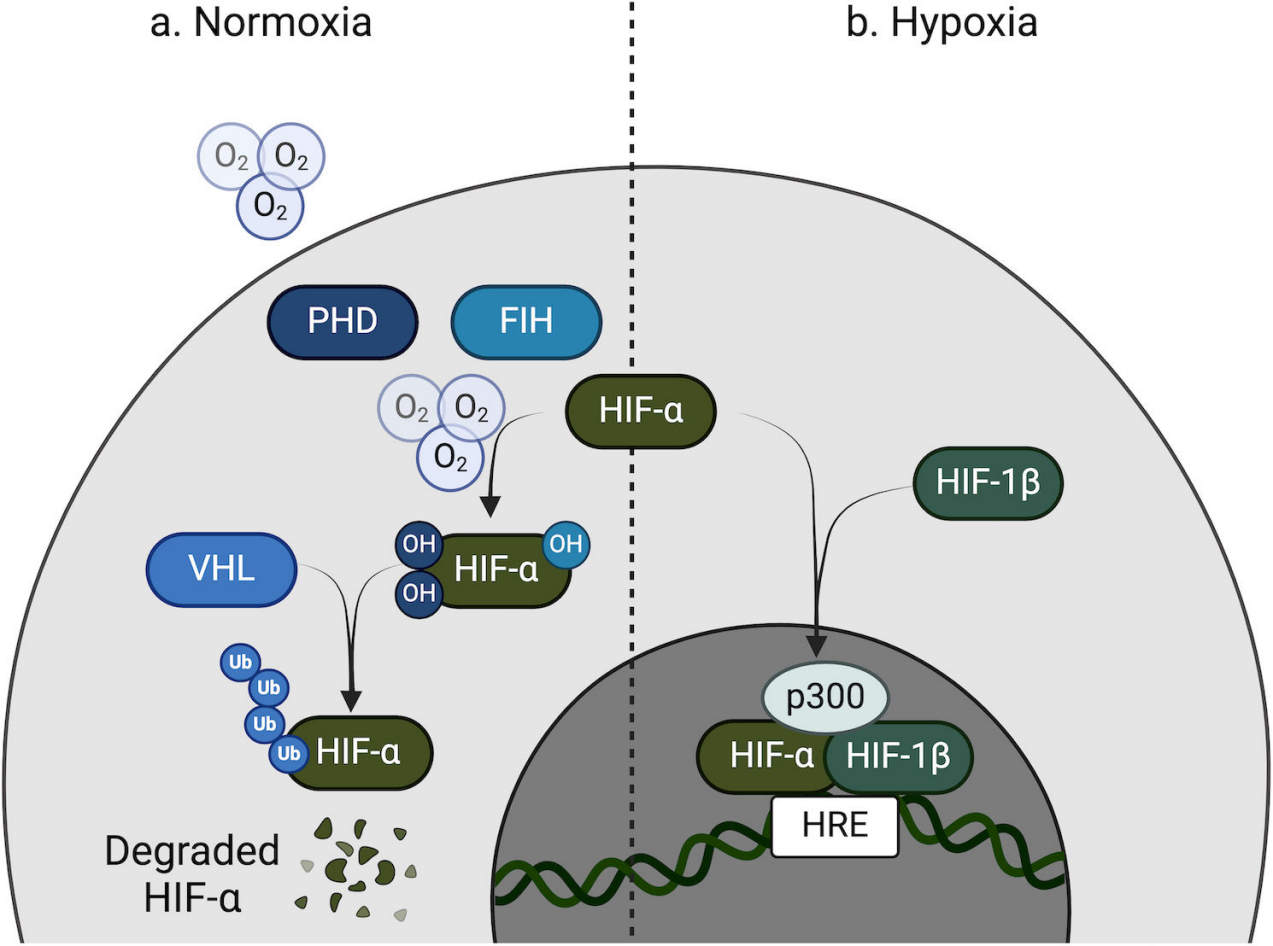
Regulation of HIF-α under normoxic and hypoxic conditions. (**a**) Normoxia: In the presence of sufficient oxygen, the oxygen-dependent enzymes, prolyl hydroxylase domain proteins and factor inhibiting HIF-1 have access to their substrate to facilitate the hydroxylation of HIF-α, rendering it inactive. When PHDs hydroxylate HIF-α, it is recognized by the VHL E3 ubiquitin ligase complex, which ubiquitinates HIF-α, targeting it for proteasomal degradation. Additionally, when FIHs hydroxylate HIF-α, it inhibits the interaction with a transcriptional co-activator, p300. (**b**) Hypoxia: Under low oxygen conditions, PHDs and FIH are inhibited due to reduced availability of oxygen as a substrate. This allows HIF-α to become stabilized, where it will translocate to the nucleus to dimerize with HIF-1β. The HIF-α/HIF-1β heterodimer recruits co-activators, such as p300, to bind HREs in the promoter regions of target genes. This interaction drives the transcription of hypoxia-responsive genes involved in processes such as angiogenesis, metabolism, and cell survival, adapting the cellular response to low oxygen levels.

The response to hypoxia is orchestrated by three HIF isoforms: HIF-1α, HIF-2α, and HIF-3α. The stability and activity of HIF-α are tightly regulated by prolyl hydroxylase domain (PHD) enzymes (7), which require oxygen, iron, and 2-oxoglutarate as essential co-factors for their function. Additionally, HIF activity is modulated by an asparaginyl hydroxylase known as factor inhibiting HIF (FIH) (9). Under normoxic conditions, PHDs hydroxylate HIF-α, marking it for recognition by the von Hippel-Lindau (VHL) ubiquitin ligase complex (8). This modification facilitates the ubiquitination and subsequent proteasomal degradation of HIF-α, effectively suppressing HIF-mediated transcriptional activity. Concurrently, FIH hydroxylates HIF-α at a distinct asparagine residue, which inhibits its interaction with the p300 co-activator, further limiting transcriptional responses (Fig. 1a). In hypoxic conditions, however, the reduced availability of oxygen inhibits both PHD and FIH activity. This allows HIF-α to stabilize, accumulate, and translocate into the nucleus, where it dimerizes with HIF-1β (also known as the aryl hydrocarbon receptor nuclear translocator, or ARNT). The HIF-α/β dimer interacts with p300 and binds to hypoxia-response elements (HREs) in the promoter regions of target genes. The activation of these genes drives critical adaptive responses to hypoxia, including angiogenesis, erythropoiesis, cell survival, cell proliferation, and metabolic reprogramming (Fig. 1b). HIF-α transcription and translation can also be regulated independent of PHD and oxygen levels by intracellular metabolites and other pathways, for example, cytokine signaling, Extracellular signal-regulated kinase (ERK)/mitogen-activated protein kinase (MAPK), and the mammalian target of rapamycin (mTOR) (10). Dysregulation of the HIF pathway is implicated in several pathological conditions, such as cancer progression, ischemic diseases, and chronic inflammation. Consequently, the HIF pathway presents a promising therapeutic target for addressing these conditions in clinical settings.

Our understanding of HIF signaling is primarily derived from studies on HIF-1α and HIF-2α, while HIF-3α is less studied (11). Despite sharing significant structural similarity and overlapping DNA binding capacities, HIF-1α and HIF-2α have distinct and nonredundant functions, largely attributed to their differential expression patterns (12, 13). HIF-1α is ubiquitously expressed across most cell types and is crucial during acute hypoxia. It shifts cellular metabolism toward anaerobic ATP production by regulating glycolytic genes, such as glucose transporter-1, and downregulating oxidative phosphorylation to reduce oxygen dependency (12, 14–17). In contrast, HIF-2α is expressed in specific cell types, including endothelial, renal, liver, cardiomyocytes, and immune cells, and plays a dominant role in chronic hypoxia, regulating genes involved in angiogenesis, erythropoiesis, and iron homeostasis (12, 18, 19). Notable HIF-2α targets include vascular endothelial growth factor (VEGF), which promotes blood vessel growth to improve oxygen delivery, and erythropoietin, which stimulates red blood cell production. Additionally, HIF-2α regulates iron homeostatic genes, such as divalent metal transporter-1 and ferroportin, to ensure adequate iron availability for erythropoiesis (19–23). These distinct functional roles highlight the complementary and time-dependent actions of HIF-1α and HIF-2α in the adaptive response to hypoxia.

Despite evolutionary adaptations to ensure adequate oxygen delivery to organs and cells, the vascularization of mammalian tissues remains highly variable. This variability results in substantial differences in oxygen availability, both between distinct tissues and within different regions of the same tissue. For example, while *in vitro* cell cultures are typically maintained at 21% oxygen—corresponding to atmospheric oxygen levels—the maximum oxygen concentration in the human body reaches only 14.5% in pulmonary alveoli (24). Much lower oxygen levels are found in tissues commonly infected by *Leishmania* parasites, such as 5% in the liver and spleen and as little as 1% in the superficial regions of the skin (25). These physiological oxygen levels, referred to as “physioxia” or tissue normoxia, vary widely depending on the organ. In contrast, hypoxia describes oxygen levels below the physiological norm for a given tissue and typically indicates insufficient oxygenation. The amount of oxygen cells are exposed to matters since optimal expression, DNA binding activity, and responses mediated by HIF-1α/β occur at oxygen levels of 1.5%–2%, reaching maximal activity at approximately 0.5% (26). In this review, we will examine studies that have used varying oxygen concentrations to assess how oxygenation impacts the ability of innate immune cells to respond to *Leishmania* infection *in vitro*. These findings are further complemented by *in vitro* and *in vivo* research utilizing animal models deficient in HIF.

### Leishmaniasis

Leishmaniases are vector-borne diseases caused by protozoan parasites from the genus *Leishmania,* with an estimated 1 million new cases occurring each year. Parasites are transmitted to the skin by the bite of an infected phlebotomine sandfly, and over 20 species cause disease in humans (27). To date, no vaccine has been approved for human use, and the available drugs are often toxic, have limited efficacy, are difficult to administer, and face increasing parasite resistance (28). Disease manifestations can range from self-healing cutaneous ulcers to disfiguring mucosal lesions and fatal visceral disease, and in all these different organs, parasites infect and survive inside cells from the innate immune system, such as macrophages, monocytes, dendritic cells, and neutrophils (29). Therefore, in order to consider novel therapeutic strategies, we must understand how our immune system can clear or sustain *Leishmania* infection.

The control of *Leishmania* requires antigen presentation by dendritic cells in the presence of co-stimulatory molecules and interleukin-12 (IL-12), leading to the differentiation of CD4 T cells into T helper 1 (Th1) cells that produce interferon-gamma (IFN-γ) and tumor necrosis factor (TNF) (30, 31). IFN-γ, in combination with TNF, activates infected cells to kill *Leishmania* by inducing the assembly of the nicotinamide adenine dinucleotide phosphate (NADPH) oxidase and the inducible or type 2 nitric oxide synthase (iNOS or NOS2) necessary for the production of reactive oxygen species (ROS) and nitric oxide (NO), respectively. These oxygen-dependent pathways are used as a defense mechanism against *Leishmania* and are required to control disease. As a successful pathogen that persists indefinitely in the mammalian host, *Leishmania* has developed mechanisms to adapt to the different intracellular and extracellular factors, recently named the “immunomicrotope” (32). One such microenvironmental factor is hypoxia. This review discusses the (i) development of hypoxia and HIF stabilization in *Leishmania* infections, followed by the effects of hypoxia and HIFs in (ii) protection and (iii) susceptibility to infection.

## DEVELOPMENT OF HYPOXIA AND HIF-Α STABILIZATION IN *LEISHMANIA* INFECTION

**Fig 2:**
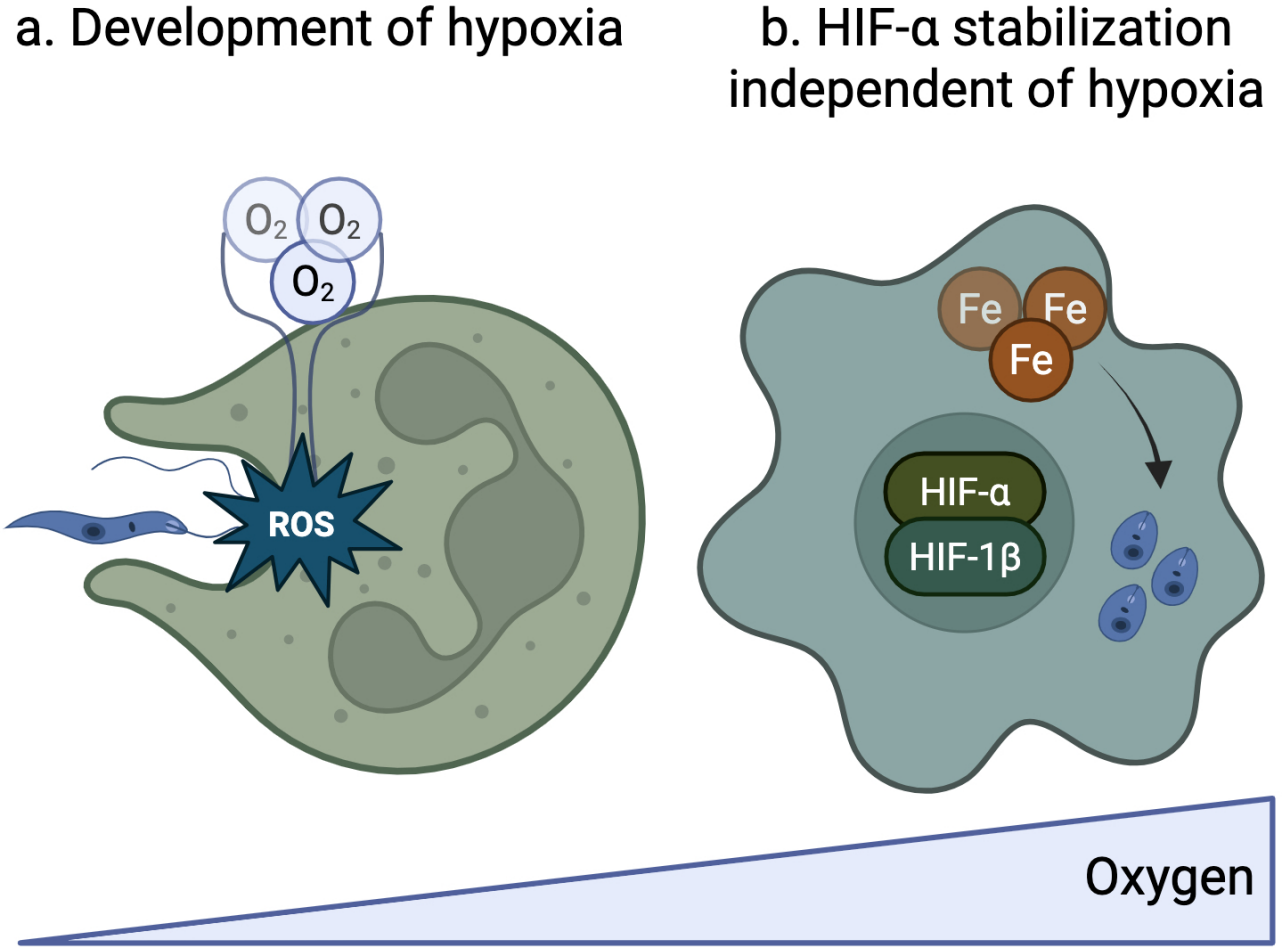
Mechanisms of HIF-α stabilization in *Leishmania* infection through hypoxia-dependent and -independent conditions. (**a**) Development of hypoxia: hypoxia can result as a by-product of immune responses to infection that require oxygen to fulfill. One major contributor to the development of hypoxia in *Leishmania* infection is through the production of ROS by neutrophils. (**b**) HIF-α stabilization independent of hypoxia: while the canonical signaling of HIF-α occurs through hypoxia, HIF-α stabilization can occur in the presence of adequate oxygen. This noncanonical signaling can be mediated through intracellular iron dysregulation facilitated by the inherent need for *Leishmania* to scavenge host cell iron to survive.

Hypoxia has been observed in cutaneous leishmaniasis lesions from human and mouse skin (33–36). Correspondingly, HIF-1α expression has been detected in both cutaneous lesions (37–39) and in the spleens of mice infected with parasites causing visceral leishmaniasis (40, 41). In cutaneous infections, lesion size inversely correlates with tissue oxygenation, with oxygen levels increasing as the lesions resolve (35). This hypoxic microenvironment appears paradoxical, given that *Leishmania*-infected skin is highly vascularized (39). Recent studies have shed light on this apparent contradiction by analyzing mRNA sequencing from human skin lesion samples. By comparing lesions with high and low hypoxic transcriptional signatures, researchers found that hypoxic lesions contained significantly more neutrophils (36). This finding suggested that neutrophils contribute to the development of hypoxia in cutaneous lesions. Subsequent mouse studies confirmed this observation, showing that while neutrophils themselves do not become hypoxic, they are surrounded by intensely hypoxic regions (36). The mechanism underlying this phenomenon was linked to the neutrophil-mediated production of ROS, which depletes local oxygen levels (36) (Fig. 2a). It is likely that other innate immune cells and oxygen-consuming pathways, such as NO production, also contribute to the hypoxic environment.

A significant consequence of hypoxia is the stabilization of HIF-α, and it is well-established that pathogens can stabilize HIF-α through mechanisms independent of hypoxia. For example, similar to *Toxoplasma gondii* (42), *Leishmania* infection of macrophages and dendritic cells induces HIF-α stabilization under normoxic conditions (43–45). One study suggests that this stabilization is thought to result not from oxygen depletion but from the parasite’s ability to manipulate host cell iron availability. In *Leishmania donovani*-infected macrophages, the depletion of intracellular iron pools inhibits PHD enzyme activity, leading to HIF-1α stabilization and conferring a survival advantage to the parasite (45) (Fig. 2b). While all *Leishmania* species depend on host iron for survival, evidence suggests that different species may employ distinct iron scavenging mechanisms, with some requiring higher iron supplies than others (46, 47). These variations could account for the differing effects of *Leishmania* infections on HIF-α stabilization. For instance, *Leishmania major*-infected macrophages did not exhibit normoxic HIF-1α stabilization, as reported by Schatz et al. Instead, they found that stabilization required additional inflammatory signals, such as IFN-γ and TNF, which are commonly present in *L. major* lesions (37). Another potential explanation for these discrepancies may result from different cells used in the experiments. While Singh et al. (45) used J774.A1 macrophage-like cell lines, studies from Schatz et al. (37) used bone-marrow-derived macrophages.

It is clear that *Leishmania* infection induces a hypoxic tissue microenvironment, and in *L. major*-infected ear pinnae, the levels of oxygen are below 0.5% (36). Furthermore, some *Leishmania* species induce HIF-α stabilization, even in normoxic conditions. The work described in the next two sections highlights studies attempting to understand how the amount of oxygen and HIF impact infection and disease outcomes in cutaneous and visceral diseases.

## PROTECTIVE ROLES OF HYPOXIA AND HIF PATHWAYS IN HOST DEFENSE AGAINST *LEISHMANIA* INFECTION

The first studies examining the impact of oxygen levels on *Leishmania*-infected macrophages investigated the effects of 5% oxygen on peritoneal macrophages and human macrophage cell lines, comparing them to atmospheric oxygen levels (21%) (48, 49). These studies found that macrophages exposed to 5% oxygen showed enhanced control of *Leishmania amazonensis* infection *in vitro* (48, 50) despite a notable 50% reduction in NO production when activated with lipopolysaccharide (LPS) and IFN-γ (48). Similarly, human dendritic cells cultured at 5% oxygen also exhibited improved control of *L. amazonensis* infection (51). The greater killing of *Leishmania* by macrophages under these low oxygen conditions was attributed to increased ROS production. This conclusion was supported by experiments utilizing the ROS scavenger *N*-acetylcysteine, which significantly increased infection rates in macrophages cultured under low oxygen conditions (50). While these studies were not conducted under true hypoxic conditions (lower than 2%), they highlighted that innate immune cells cultured in physioxia (e.g., 5% oxygen) demonstrate an enhanced ability to control *Leishmania* parasites *in vitro* compared to cells maintained at ambient oxygen levels. These findings emphasize the importance of physiological oxygen levels in shaping the antimicrobial functions of innate immune cells.

Building on the role of low oxygen in modulating macrophage function, studies have explored the specific involvement of HIF-1α in the control of *Leishmania* infection. While pharmacological stabilization of HIF-1α alone does not appear to enhance the microbicidal activity of macrophages, its expression in *L. major*-infected macrophages activated by LPS and IFN-γ is crucial for controlling *Leishmania in vitro* (37). *In vivo* studies further demonstrated that myeloid-specific deletion of HIF-1α using Lysm^cre^Hif1a^flox/flox^ mice leads to reduced iNOS expression in myeloid cells, resulting in increased parasite loads and larger lesions during the later stages of the disease (37). Similarly, HIF-1α-dependent induction of NADPH oxidase was shown to be necessary for adequate ROS production in *L. amazonensis*-infected macrophages cultured in 3% oxygen (52). Collectively, these data suggest that HIF-1α expression is important to mediate iNOS and NADPH oxidase induction in myeloid cells and control cutaneous leishmaniasis caused by *L. major* and *L. amazonensis*.

Evidence suggests that HIF-1β expression in myeloid cells is crucial for the production of VEGF-A during *L. major* infection. Myeloid-specific deficiency of HIF-1β results in larger lesions without affecting parasite numbers in the infected skin (53). These findings align with earlier studies demonstrating that VEGF-A deletion increases lesion size while having no impact on parasite control (39). The observed increase in lesion size in myeloid HIF-1β-deficient mice is attributed to altered vascular remodeling, which leads to an accumulation of monocytes and Th1 cells in the lesions due to reduced VEGF-A expression (53, 54). In addition to HIF-α isoforms, HIF-1β is known to dimerize with various proteins, and each dimeric complex interacts with specific DNA elements to regulate distinct gene expression pathways (55). Therefore, it is unknown which specific binding partners are responsible for the observed inflammatory effects and increased lesion size in animals with myeloid-specific deletion of HIF-1β. Identifying those specific binding partners will help clarify whether these effects are directly related to the hypoxic environment of cutaneous leishmaniasis lesions caused by *L. major* and/or involve other pathways.

HIF-1α also plays a protective role against *L. donovani* infection by regulating macrophage metabolism. In Lysm^cre^Hif1a^flox/flox^ mice infected with *L. donovani*, the loss of HIF-1α resulted in sustained activation of the mTOR pathway, which contributed to increased fatty acid synthesis (41). This dysregulated lipid metabolism rendered mice more susceptible to *L. donovani* infection. Similarly, individuals carrying a single-nucleotide polymorphism that reduces *HIF1A* expression exhibit increased lipogenesis and are more susceptible to *L. donovani* infection *in vitro* (41). These data support a role for HIF-1α in the ability of myeloid cells to suppress fatty acid synthesis and control *Leishmania* species that cause visceral leishmaniasis.

**Fig 3:**
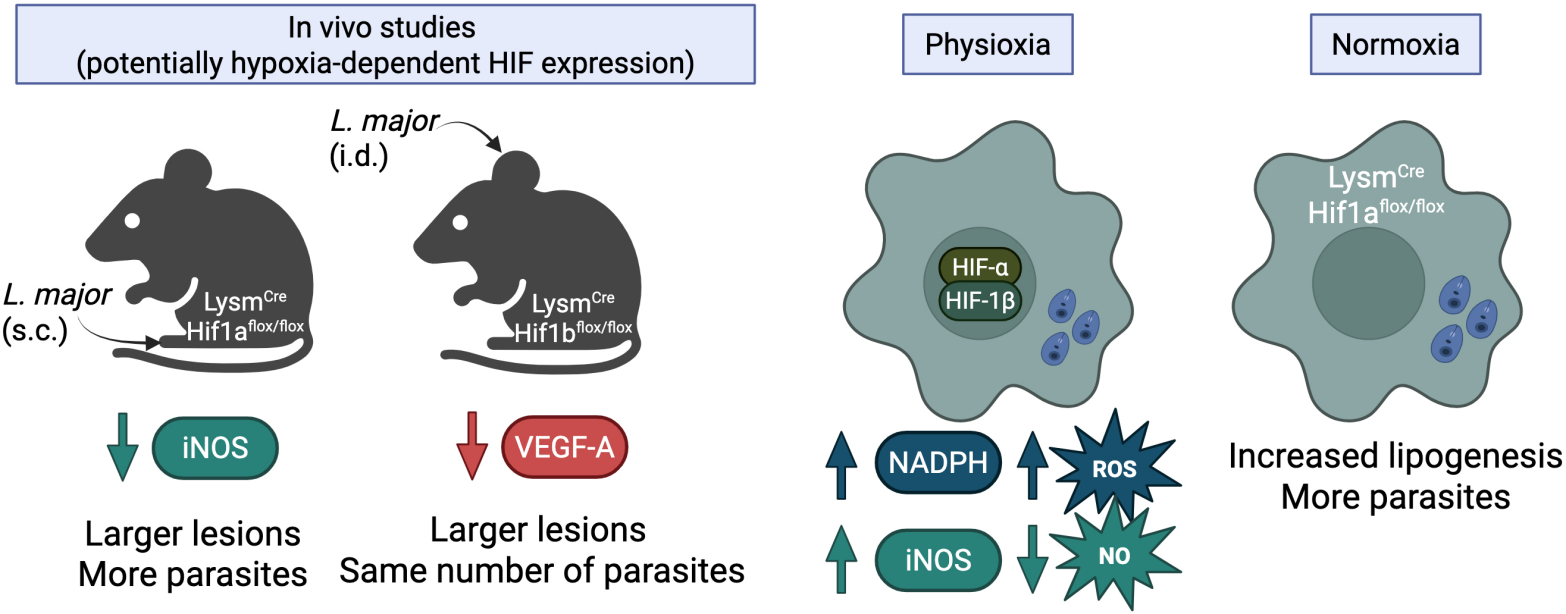
Role of HIF-α in host defense to *Leishmania* infection under hypoxic, physioxic, and normoxic conditions. *In vivo* studies (potentially hypoxia-dependent HIF stabilization): mice with myeloid-specific HIF-1α deletion (Lysm^Cre^Hif1a^fl/fl^) infected with *L. major* subcutaneously (s.c.) exhibit larger lesions and increased parasite burdens due to reduced iNOS expression. Additionally, mice with myeloid-specific HIF-1β deletion (Lysm^Cre^Hif1b^fl/fl^) infected intradermally (i.d.) with *L. major* develop larger lesions, but the parasite burden remains unchanged. This is attributed to increased VEGF-A production, which exacerbates lesion growth through changes in vascularization but does not affect parasite replication. Physioxia: in *Leishmania*-infected cells cultured at physiological oxygen levels (5% oxygen), HIF-1α/HIF-1β stabilization enhances NADPH oxidase production, leading to increased ROS production and enhances iNOS expression, but this does not confer to higher NO levels as infected macrophages show diminished NO production. Normoxia: macrophages with HIF-1α deficiency (Lysm^Cre^Hif1a^fl/fl^) exhibit increased lipogenesis, which allows for a greater survival advantage to support parasite replication, suggesting that HIF-1α plays a protective role in restricting parasite growth under normal oxygen levels.

Collectively, these studies highlight the importance of including low oxygen conditions when evaluating the ability of cells to control pathogens (Fig. 3). Furthermore, these studies suggest that HIF expression in myeloid cells can bolster host defenses against *Leishmania* infections. However, there is also substantial contrasting evidence that low oxygen tensions (5%), hypoxia (oxygen tensions below 2%), and HIF expression in immune cells play a significant role in promoting disease progression, which may be dependent or independent of the parasite burden, and which will be explored in the following section.

## HYPOXIA AND HIF PATHWAYS IN HOST SUSCEPTIBILITY AND DISEASE PROGRESSION IN *LEISHMANIA* INFECTIONS

Low oxygen significantly impacts the immune response to *L. major and L. amazonensis* infection by altering the production of key antimicrobial molecules such as NO. While HIF-1α was shown to be important for iNOS expression (37), earlier studies by the same group showed that NO production is lower in macrophages cultured in hypoxia (0.5%–2% oxygen) (35). Experiments with *L. amazonensis* also showed a reduction in NO production in mouse and human macrophages cultured under 5% oxygen tension (48). Conversely, exposing *L. amazonensis*-infected macrophages to hyperoxic conditions in a hyperbaric chamber enhanced parasite control (56). However, it remains unclear whether this effect resulted from improved macrophage antimicrobial activity in a high-oxygen environment or a direct inhibitory impact of hyperoxia on *L. amazonensis* survival. Other *in vivo* studies provide additional clarity on the role of hypoxia in *Leishmania* infections. Wild-type mice exposed to systemic hypoxia exhibited reduced tissue oxygenation in cutaneous lesions, impairing parasite control (35). Interestingly, in iNOS-deficient mice, parasite loads were similar under normoxic and hypoxic conditions, demonstrating that hypoxia primarily undermines *Leishmania* control by depriving iNOS of the oxygen required for NO production (35). These findings reveal a critical limitation of the host’s ability to control *Leishmania* infections in hypoxic conditions, where insufficient oxygen availability hampers the production of NO, providing a survival advantage to the parasite.

In visceral leishmaniasis, Hammami et al. demonstrated that HIF-1α in dendritic cells supports *L. donovani* infection *in vivo*. Using CD11c^cre^Hif1a^flox/flox^ mice, they showed that dendritic cells deficient in HIF-1α produced higher levels of IL-12 (40, 57), a cytokine critical for anti-parasitic immunity. Supporting this observation, human dendritic cells and macrophages infected with *L. amazonensis* produced significantly less IL-12 when cultured under 5% oxygen tension compared to normoxia (50, 51). Additionally, HIF-1α-deficient dendritic cells produced reduced levels of the regulatory cytokine IL-10, which is known to dampen immune responses (40, 57). This dual effect of HIF-1α—suppressing IL-12 while promoting IL-10—renders dendritic cells less effective in supporting T cell-mediated control of infection. As a consequence of the ablation of HIF-1α in dendritic cells, the authors observed enhanced development of short-lived effector CD8 T cells (40) and increased frequency of CD4 T cells producing IFN-γ (57), both of which are important for the control of visceral disease. Moreover, HIF-1α was implicated in the expansion of myeloid-derived suppressor cells in the spleens of *L. donovani*-infected mice, further increasing susceptibility to infection (58). Supporting these findings, pharmacological inhibition of HIF-1α *in vivo* significantly reduces parasite burden in the liver and spleen of infected mice (59) and suggests a detrimental role of HIF-1α in visceral disease. Taken together, these findings suggest that low oxygen levels and HIF-1α play a detrimental role in innate immunity during visceral leishmaniasis by impairing the functionality of dendritic cells and promoting immunosuppressive pathways in T cells. The discrepancy between these findings and studies showing that HIF-1α protects against *L. donovani* by modulating fatty acid synthesis remains unclear but may be explained by differences in the cell subsets studied (e.g., macrophages versus dendritic cells). Further investigation is needed to clarify this divergence.

**Fig 4:**
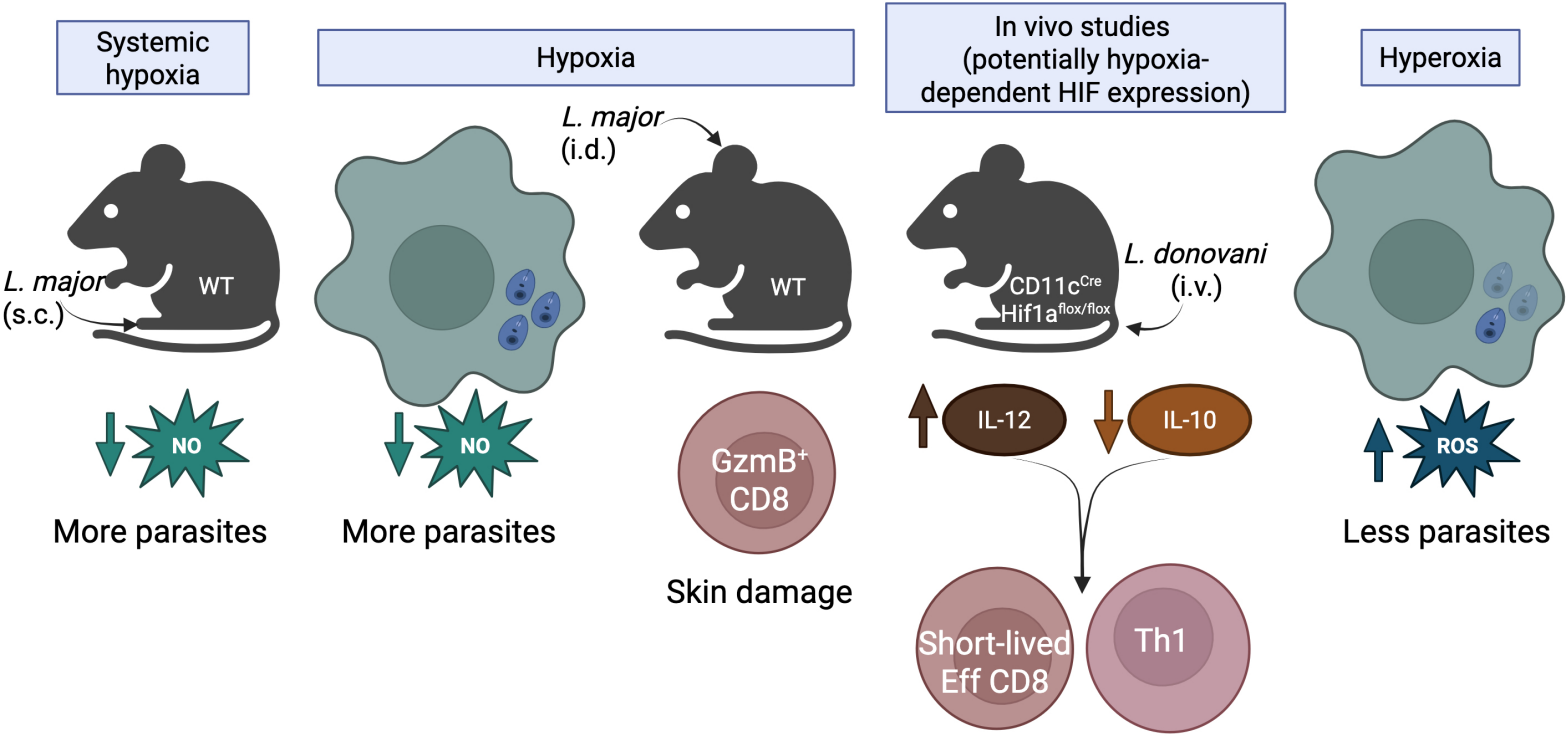
Effects of oxygen levels and HIF signaling on host susceptibility during *Leishmania* infection. Systemic hypoxia: wild-type (WT) mice infected subcutaneously (s.c.) with *L. major* exposed to systemic hypoxia exhibit a reduction in NO due to a decrease in oxygen levels needed for iNOS to turnover NO production. This dampening of NO leads to an increase in parasite burdens, as it is a critical effector molecule for *Leishmania* control. Hypoxia: *in vitro* studies demonstrate that hypoxic conditions in macrophages reduce NO production, impairing the ability to control *L. major* infection. Additionally, intradermal (i.d.) infection of WT mice with *L. major* creates highly inflamed and hypoxic regions. CD8 T cells that have newly migrated from the draining lymph node to the hypoxic skin upregulate granzyme B production. These granzyme B-positive CD8 T cells do not control parasites and only exacerbate inflammation and delay wound healing. *In vivo* studies (potentially hypoxia-dependent HIF stabilization): mice with dendritic cell-specific deletion of HIF-1α (CD11c^Cre^ Hif1a^fl/fl^) infected intravenously (i.v.) with *L. donovani* exhibit altered cytokine profiles, including increased IL-12 and decreased IL-10 production. This cytokine shift promotes the generation of short-lived effector (Eff) CD8 T cell and CD4 Th1 cell responses, which are both necessary for the control of visceral disease. This study highlights the impact of HIF-1α signaling in dendritic cells, where it controls the production of IL-10 while dampening IL-12 production, which benefits *Leishmania* survival and pro-inflammatory immune evasion. Hyperoxia: *in vitro* studies of *Leishmania*-infected macrophages cultured under hyperoxic conditions show increased ROS production, leading to enhanced parasite killing. These data suggest that high oxygen levels favor parasite clearance, whereas low oxygen levels provide a greater opportunity for parasite survival.

While the studies in visceral leishmaniasis using CD11c^cre^Hif1a^flox/flox^ mice suggest that hypoxia indirectly suppresses T cell responses by affecting dendritic cell function, hypoxia can also directly influence the fate and function of CD4 and CD8 T cells (60–77). To date, only one study has examined the direct impact of hypoxia on T cells in the context of leishmaniasis. This study demonstrated that the hypoxic environment of cutaneous leishmaniasis lesions during *L. major* and *Leishmania braziliensis* infections promotes the expression of the cytotoxic molecule granzyme B in CD8 T cells (36). Mechanistically, CD8 T cells are initially activated in draining lymph nodes, where oxygen levels are relatively higher. Once activated, these cells migrate to inflamed, hypoxic skin lesions with oxygen tensions below 0.5%. The hypoxic microenvironment reprograms CD8 T cells, driving the upregulation of granzyme B. There is ample evidence that cytotoxic CD8 T cells damage the *Leishmania*-infected skin, delay wound healing, and do not effectively control *Leishmania* infection (78–88). Thus, hypoxia skews CD8 T cell responses toward a phenotype that is detrimental to the host, highlighting the negative impact of hypoxia on adaptive immune responses in cutaneous leishmaniasis (Fig. 4).

## CONCLUSIONS AND OPEN QUESTIONS

The role of low oxygen, hypoxia, and HIFs in leishmaniasis is complex and varies depending on factors such as the *Leishmania* species, affected cell types, mouse models used, and the degree of oxygenation (Table 1). While it is tempting to categorize microenvironmental signals and transcription factors as either beneficial or detrimental, the reality is likely more nuanced. For instance, as demonstrated by Fowler et al. (36), regions of severe hypoxia, such as those with high neutrophil density, may deprive infected macrophages of molecular oxygen necessary for NO production, impairing their ability to kill *Leishmania*, even if iNOS expression is elevated by HIF-1α stabilization. In contrast, macrophages in moderately hypoxic regions of the same lesion may benefit from iNOS expression induced by HIF-1α stabilization, with sufficient molecular oxygen available to sustain NO production and effectively control the infection. Similarly, immune cells closer to blood vessels may not experience severe hypoxia and thus function differently within the same microenvironment. To better understand these dynamics, future studies should focus on mapping hypoxia distribution and immune cell subsets within infected tissues. Integrating this with research conducted under varying oxygen pressures will provide deeper insights into the differential outcomes driven by hypoxia in leishmaniasis.

**TABLE 1:** Summary of studies on oxygen levels, HIF deletion, and the impact on protection or susceptibility to *Leishmania* infection

*Leishmania* species	Clinical presentation	Models studied	Host response	References	
				Low oxygen	HIF deletion
*L. major*	Cutaneous	Mouse	Protection	(37)	(37, 53, 54)
			Susceptibility	(35, 36)	None
*L. braziliensis*	Cutaneous	Mouse and human	Protection	None	None
			Susceptibility	(36)	None
*L. amazonensis*	Cutaneous	Mouse and human	Protection	(48–52)	(52)
			Susceptibility	(56)	None
*L. donovani*	Visceral	Mouse and human	Protection	None	(41)
			Susceptibility	None	(40, 57–59)

The existing research underscores the significant role of oxygen levels and HIFs in shaping the immune response, particularly focusing on immune cells. However, it is evident that all cells within the hypoxic microenvironment of infected tissues, not just immune cells, are influenced by oxygen deprivation. Epithelial cells, for instance, are well-known for their ability to orchestrate immune responses and react to inflammatory signals (89). While they do not express iNOS under hypoxic conditions (90), they may still be directly or indirectly impacted by low oxygen (91–93). Yet, how epithelial cells respond to hypoxia and how this response might influence disease resolution in leishmaniasis remain largely unexplored, representing an important avenue for future research.

One understudied aspect of hypoxia in *Leishmania* infection is its direct impact on the parasites. *Leishmania* is likely exposed to hypoxic conditions both within the sandfly vector and in the mammalian host. However, our understanding of how *Leishmania* adapts to low oxygen environments remains limited (94). Investigating the cellular and molecular mechanisms that enable the parasite to survive and thrive under hypoxia is crucial for advancing our knowledge of *Leishmania* biology and potentially identifying novel therapeutic targets.

Another crucial area of research is exploring how hypoxia influences the host’s ability to respond to therapy. Studies have shown a modest but significant reduction in the efficacy of anti-parasitic drugs under hypoxic conditions *in vitro* (95), highlighting the need to consider hypoxia as a key microenvironmental factor when developing and testing novel therapeutics. Separately, studies in mice deficient in ROS and NO production have shown impaired control of *L. donovani* following treatment with anti-parasitic drugs (96). Since ROS and NO production require oxygen, these mice are expected to experience reduced levels of tissue hypoxia. While the Murray et al. study (96) did not directly evaluate the hypoxic state of infected tissues, these findings highlight that factors beyond hypoxia may also critically influence drug efficacy. Collectively, these observations underscore the complexity of the tissue microenvironment and the importance of integrating multiple host factors, including oxygen availability, into the design of future drug studies to optimize treatment outcomes.

A key challenge in this field is the inconsistent use of oxygen definitions, with many studies employing 5%–6% oxygen as a model for hypoxia—despite this level aligning more closely with physioxia than true hypoxia. This variability complicates comparisons across studies and highlights the need for standardized definitions for those studying hypoxia in leishmaniasis. Despite these challenges, this review has highlighted significant progress in understanding the role of oxygen levels as a critical component of the immunomicrotope in *Leishmania*-induced disease. It also emphasizes how much remains unknown about this dynamic process, presenting an exciting and expansive field for future research. By addressing these gaps, we can deepen our understanding of hypoxia’s dual role in infection and therapy, paving the way for novel insights and transformative advancements in leishmaniasis research.

## References

[1] Raymond J, Segrè D. 2006. The effect of oxygen on biochemical networks and the evolution of complex life. Science 311:1764–1767. doi:10.1126/science.1118439 16556842

[2] Baik AH, Jain IH. 2020. Turning the oxygen dial: balancing the highs and lows. Trends Cell Biol 30:516–536. doi:10.1016/j.tcb.2020.04.005 32386878 PMC7391449

[3] Warburg O. 1956. On the origin of cancer cells. Science. 10.1126/science.123.3191.309 13298683

[4] Semenza GL, Nejfelt MK, Chi SM, Antonarakis SE. 1991. Hypoxia-inducible nuclear factors bind to an enhancer element located 3’ to the human erythropoietin gene. Proc Natl Acad Sci USA 88:5680–5684. doi:10.1073/pnas.88.13.5680 2062846 PMC51941

[5] Wang GL, Jiang BH, Rue EA, Semenza GL. 1995. Hypoxia-inducible factor 1 is a basic-helix-loop-helix-PAS heterodimer regulated by cellular O2 tension. Proc Natl Acad Sci USA 92:5510–5514. doi:10.1073/pnas.92.12.5510 7539918 PMC41725

[6] Maxwell PH, Wiesener MS, Chang GW, Clifford SC, Vaux EC, Cockman ME, Wykoff CC, Pugh CW, Maher ER, Ratcliffe PJ. 1999. The tumour suppressor protein VHL targets hypoxia-inducible factors for oxygen-dependent proteolysis. Nature 399:271–275. doi:10.1038/20459 10353251

[7] Ivan M, Kondo K, Yang H, Kim W, Valiando J, Ohh M, Salic A, Asara JM, Lane WS, Kaelin WG. 2001. HIFα targeted for VHL-mediated destruction by proline hydroxylation: implications for O_2_ sensing. Science 292:464–468. doi:10.1126/science.1059817 11292862

[8] Jaakkola P, Mole DR, Tian YM, Wilson MI, Gielbert J, Gaskell SJ, von Kriegsheim A, Hebestreit HF, Mukherji M, Schofield CJ, Maxwell PH, Pugh CW, Ratcliffe PJ. 2001. Targeting of HIF-α to the von Hippel-Lindau ubiquitylation complex by O_2_-regulated prolyl hydroxylation. Science 292:468–472. doi:10.1126/science.1059796 11292861

[9] Mahon PC, Hirota K, Semenza GL. 2001. FIH-1: a novel protein that interacts with HIF-1α and VHL to mediate repression of HIF-1 transcriptional activity. Genes Dev 15:2675–2686. doi:10.1101/gad.924501 11641274 PMC312814

[10] Lee P, Chandel NS, Simon MC. 2020. Cellular adaptation to hypoxia through hypoxia inducible factors and beyond. Nat Rev Mol Cell Biol 21:268–283. doi:10.1038/s41580-020-0227-y 32144406 PMC7222024

[11] Duan C. 2016. Hypoxia-inducible factor 3 biology: complexities and emerging themes. Am J Physiol Cell Physiol 310:C260–9. doi:10.1152/ajpcell.00315.2015 26561641

[12] Hu C-J, Wang L-Y, Chodosh LA, Keith B, Simon MC. 2003. Differential roles of hypoxia-inducible factor 1α (HIF-1α) and HIF-2α in hypoxic gene regulation. Mol Cell Biol 23:9361–9374. doi:10.1128/MCB.23.24.9361-9374.2003 14645546 PMC309606

[13] Keith B, Johnson RS, Simon MC. 2011. HIF1α and HIF2α: sibling rivalry in hypoxic tumour growth and progression. Nat Rev Cancer 12:9–22. doi:10.1038/nrc3183 22169972 PMC3401912

[14] Papandreou I, Cairns RA, Fontana L, Lim AL, Denko NC. 2006. HIF-1 mediates adaptation to hypoxia by actively downregulating mitochondrial oxygen consumption. Cell Metab 3:187–197. doi:10.1016/j.cmet.2006.01.012 16517406

[15] Semenza GL, Jiang BH, Leung SW, Passantino R, Concordet JP, Maire P, Giallongo A. 1996. Hypoxia response elements in the aldolase A, enolase 1, and lactate dehydrogenase A gene promoters contain essential binding sites for hypoxia-inducible factor 1. J Biol Chem 271:32529–32537. doi:10.1074/jbc.271.51.32529 8955077

[16] Semenza GL, Roth PH, Fang HM, Wang GL. 1994. Transcriptional regulation of genes encoding glycolytic enzymes by hypoxia-inducible factor 1. J Biol Chem 269:23757–23763. doi:10.1016/S0021-9258(17)31580-6 8089148

[17] Ebert BL, Firth JD, Ratcliffe PJ. 1995. Hypoxia and mitochondrial inhibitors regulate expression of glucose transporter-1 via distinct Cis-acting sequences. J Biol Chem 270:29083–29089. doi:10.1074/jbc.270.49.29083 7493931

[18] Koh MY, Lemos R, Liu X, Powis G. 2011. The hypoxia-associated factor switches cells from HIF-1α- to HIF-2α-dependent signaling promoting stem cell characteristics, aggressive tumor growth and invasion. Cancer Res 71:4015–4027. doi:10.1158/0008-5472.CAN-10-4142 21512133 PMC3268651

[19] Rankin EB, Biju MP, Liu Q, Unger TL, Rha J, Johnson RS, Simon MC, Keith B, Haase VH. 2007. Hypoxia-inducible factor-2 (HIF-2) regulates hepatic erythropoietin in vivo. J Clin Invest 117:1068–1077. doi:10.1172/JCI30117 17404621 PMC1838939

[20] Rankin EB, Rha J, Unger TL, Wu CH, Shutt HP, Johnson RS, Simon MC, Keith B, Haase VH. 2008. Hypoxia-inducible factor-2 regulates vascular tumorigenesis in mice. Oncogene 27:5354–5358. doi:10.1038/onc.2008.160 18490920 PMC2575082

[21] Mastrogiannaki M, Matak P, Keith B, Simon MC, Vaulont S, Peyssonnaux C. 2009. HIF-2α, but not HIF-1α, promotes iron absorption in mice. J Clin Invest 119:1159–1166. doi:10.1172/JCI38499 19352007 PMC2673882

[22] Shah YM, Matsubara T, Ito S, Yim S-H, Gonzalez FJ. 2009. Intestinal hypoxia-inducible transcription factors are essential for iron absorption following iron deficiency. Cell Metab 9:152–164. doi:10.1016/j.cmet.2008.12.012 19147412 PMC2659630

[23] Taylor M, Qu A, Anderson ER, Matsubara T, Martin A, Gonzalez FJ, Shah YM. 2011. Hypoxia-inducible factor-2α mediates the adaptive increase of intestinal ferroportin during iron deficiency in mice. Gastroenterology 140:2044–2055. doi:10.1053/j.gastro.2011.03.007 21419768 PMC3109109

[24] Mirchandani AS, Sanchez-Garcia MA, Walmsley SR. 2025. How oxygenation shapes immune responses: emerging roles for physioxia and pathological hypoxia. Nat Rev Immunol 25:161–177. doi:10.1038/s41577-024-01087-5 39349943

[25] Dietrich P-Y, Walker PR. 2016. Hypoxia and antitumor CD8^+^ T cells: an incompatible alliance? Oncoimmunology 5:e1232236. doi:10.1080/2162402X.2016.1232236 28123871 PMC5214994

[26] Jiang BH, Semenza GL, Bauer C, Marti HH. 1996. Hypoxia-inducible factor 1 levels vary exponentially over a physiologically relevant range of O2 tension. Am J Physiol 271:C1172–C1180. doi:10.1152/ajpcell.1996.271.4.C1172 8897823

[27] Kaye P, Scott P. 2011. Leishmaniasis: complexity at the host-pathogen interface. Nat Rev Microbiol 9:604–615. doi:10.1038/nrmicro2608 21747391

[28] Novais FO, Amorim CF, Scott P. 2021. Host-directed therapies for cutaneous leishmaniasis. Front Immunol 12:660183. doi:10.3389/fimmu.2021.660183 33841444 PMC8032888

[29] Scott P, Novais FO. 2016. Cutaneous leishmaniasis: immune responses in protection and pathogenesis. Nat Rev Immunol 16:581–592. doi:10.1038/nri.2016.72 27424773

[30] Heinzel FP, Schoenhaut DS, Rerko RM, Rosser LE, Gately MK. 1993. Recombinant interleukin 12 cures mice infected with Leishmania major. J Exp Med 177:1505–1509. doi:10.1084/jem.177.5.1505 8097524 PMC2191017

[31] Scott P, Natovitz P, Coffman RL, Pearce E, Sher A. 1988. Immunoregulation of cutaneous leishmaniasis. T cell lines that transfer protective immunity or exacerbation belong to different T helper subsets and respond to distinct parasite antigens. J Exp Med 168:1675–1684. doi:10.1084/jem.168.5.1675 2903212 PMC2189120

[32] Bogdan C, Islam N-A-K, Barinberg D, Soulat D, Schleicher U, Rai B. 2024. The immunomicrotope of Leishmania control and persistence. Trends Parasitol 40:788–804. doi:10.1016/j.pt.2024.07.013 39174373

[33] Werth N, Beerlage C, Rosenberger C, Yazdi AS, Edelmann M, Amr A, Bernhardt W, von Eiff C, Becker K, Schäfer A, Peschel A, Kempf VAJ. 2010. Activation of hypoxia inducible factor 1 is a general phenomenon in infections with human pathogens. PLoS One 5:e11576. doi:10.1371/journal.pone.0011576 20644645 PMC2904385

[34] Araújo AP, Arrais-Silva WW, Giorgio S. 2012. Infection by Leishmania amazonensis in mice: a potential model for chronic hypoxia. Acta Histochem 114:797–804. doi:10.1016/j.acthis.2012.01.007 22360823

[35] Mahnke A, Meier RJ, Schatz V, Hofmann J, Castiglione K, Schleicher U, Wolfbeis OS, Bogdan C, Jantsch J. 2014. Hypoxia in Leishmania major skin lesions impairs the NO-dependent leishmanicidal activity of macrophages. J Invest Dermatol 134:2339–2346. doi:10.1038/jid.2014.121 24583949

[36] Fowler EA, Farias Amorim C, Mostacada K, Yan A, Amorim Sacramento L, Stanco RA, Hales ED, Varkey A, Zong W, Wu GD, de Oliveira CI, Collins PL, Novais FO. 2024. Neutrophil-mediated hypoxia drives pathogenic CD8^+^ T cell responses in cutaneous leishmaniasis. J Clin Invest 134:e177992. doi:10.1172/JCI177992 38833303 PMC11245163

[37] Schatz V, Strüssmann Y, Mahnke A, Schley G, Waldner M, Ritter U, Wild J, Willam C, Dehne N, Brüne B, McNiff JM, Colegio OR, Bogdan C, Jantsch J. 2016. Myeloid cell-derived HIF-1α promotes control of Leishmania major. J Immunol 197:4034–4041. doi:10.4049/jimmunol.1601080 27798163 PMC7249608

[38] Arrais-Silva WW, Paffaro VA, Yamada AT, Giorgio S. 2005. Expression of hypoxia-inducible factor-1α in the cutaneous lesions of BALB/c mice infected with Leishmania amazonensis. Exp Mol Pathol 78:49–54. doi:10.1016/j.yexmp.2004.09.002 15596060

[39] Weinkopff T, Konradt C, Christian DA, Discher DE, Hunter CA, Scott P. 2016. Leishmania major infection-induced VEGF-A/VEGFR-2 signaling promotes lymphangiogenesis that controls disease. J Immunol 197:1823–1831. doi:10.4049/jimmunol.1600717 27474074 PMC5001553

[40] Hammami A, Charpentier T, Smans M, Stäger S. 2015. IRF-5-mediated inflammation limits CD8^+^ T cell expansion by inducing HIF-1α and impairing dendritic cell functions during Leishmania infection. PLoS Pathog 11:e1004938. doi:10.1371/journal.ppat.1004938 26046638 PMC4457842

[41] Mesquita I, Ferreira C, Moreira D, Kluck GEG, Barbosa AM, Torrado E, Dinis-Oliveira RJ, Gonçalves LG, Beauparlant C-J, Droit A, Berod L, Sparwasser T, Bodhale N, Saha B, Rodrigues F, Cunha C, Carvalho A, Castro AG, Estaquier J, Silvestre R. 2020. The absence of HIF-1α increases susceptibility to Leishmania donovani infection via activation of BNIP3/mTOR/SREBP-1c axis. Cell Rep 30:4052–4064. doi:10.1016/j.celrep.2020.02.098 32209468

[42] Spear W, Chan D, Coppens I, Johnson RS, Giaccia A, Blader IJ. 2006. The host cell transcription factor hypoxia-inducible factor 1 is required for Toxoplasma gondii growth and survival at physiological oxygen levels. Cell Microbiol 8:339–352. doi:10.1111/j.1462-5822.2005.00628.x 16441443

[43] Degrossoli A, Bosetto MC, Lima CBC, Giorgio S. 2007. Expression of hypoxia-inducible factor 1α in mononuclear phagocytes infected with Leishmania amazonensis. Immunol Lett 114:119–125. doi:10.1016/j.imlet.2007.09.009 17983667

[44] Kumar V, Kumar A, Das S, Kumar A, Abhishek K, Verma S, Mandal A, Singh RK, Das P. 2018. Leishmania donovani activates hypoxia inducible factor-1α and miR-210 for survival in macrophages by downregulation of NF-κB mediated pro-inflammatory immune response. Front Microbiol 9:385. doi:10.3389/fmicb.2018.00385 29568285 PMC5852103

[45] Singh AK, Mukhopadhyay C, Biswas S, Singh VK, Mukhopadhyay CK. 2012. Intracellular pathogen Leishmania donovani activates hypoxia inducible factor-1 by dual mechanism for survival advantage within macrophage. PLoS One 7:e38489. doi:10.1371/journal.pone.0038489 22701652 PMC3373497

[46] Zaidi A, Singh KP, Ali V. 2017. Leishmania and its quest for iron: an update and overview. Mol Biochem Parasitol 211:15–25. doi:10.1016/j.molbiopara.2016.12.004 27988301

[47] Goto Y, Ito T, Ghosh S, Mukherjee B. 2023. Access and utilization of host-derived iron by Leishmania parasites. J Biochem 175:17–24. doi:10.1093/jb/mvad082 37830941 PMC10771036

[48] Colhone MC, Arrais-Silva WW, Picoli C, Giorgio S. 2004. Effect of hypoxia on macrophage infection by Leishmania amazonensis. J Parasitol 90:510–515. doi:10.1645/GE-3286 15270094

[49] Degrossoli A, Colhone MC, Arrais-Silva WW, Giorgio S. 2004. Hypoxia modulates expression of the 70-kD heat shock protein and reduces Leishmania infection in macrophages. J Biomed Sci 11:847–854. doi:10.1007/BF02254370 15591782

[50] Degrossoli A, Arrais-Silva WW, Colhone MC, Gadelha FR, Joazeiro PP, Giorgio S. 2011. The influence of low oxygen on macrophage response to Leishmania infection. Scand J Immunol 74:165–175. doi:10.1111/j.1365-3083.2011.02566.x 21517930

[51] Bosseto MC, Palma PVB, Covas DT, Giorgio S. 2010. Hypoxia modulates phenotype, inflammatory response, and leishmanial infection of human dendritic cells. APMIS 118:108–114. doi:10.1111/j.1600-0463.2009.02568.x 20132174

[52] Alonso D, Serrano E, Bermejo FJ, Corral RS. 2019. HIF-1α-regulated MIF activation and Nox2-dependent ROS generation promote Leishmania amazonensis killing by macrophages under hypoxia. Cell Immunol 335:15–21. doi:10.1016/j.cellimm.2018.10.007 30384962

[53] Weinkopff T, Roys H, Bowlin A, Scott P. 2019. Leishmania infection induces macrophage vascular endothelial growth factor A production in an ARNT/HIF-dependent manner. Infect Immun 87:e00088-19. doi:10.1128/IAI.00088-19 31451620 PMC6803331

[54] Bowlin A, Roys H, Wanjala H, Bettadapura M, Venugopal G, Surma J, Simon MC, Weinkopff T. 2021. Hypoxia-inducible factor signaling in macrophages promotes lymphangiogenesis in Leishmania major infection. Infect Immun 89:e0012421. doi:10.1128/IAI.00124-21 34031127 PMC8281282

[55] Mandl M, Depping R. 2014. Hypoxia-inducible aryl hydrocarbon receptor nuclear translocator (ARNT) (HIF-1β): is it a rare exception? Mol Med 20:215–220. doi:10.2119/molmed.2014.00032 24849811 PMC4069271

[56] Arrais-Silva WW, Collhone MC, Ayres DC, de Souza Souto PC, Giorgio S. 2005. Effects of hyperbaric oxygen on Leishmania amazonensis promastigotes and amastigotes. Parasitol Int 54:1–7. doi:10.1016/j.parint.2004.07.002 15710544

[57] Hammami A, Abidin BM, Heinonen KM, Stäger S. 2018. HIF-1α hampers dendritic cell function and Th1 generation during chronic visceral leishmaniasis. Sci Rep 8:3500. doi:10.1038/s41598-018-21891-z 29472618 PMC5823892

[58] Hammami A, Abidin BM, Charpentier T, Fabié A, Duguay A-P, Heinonen KM, Stäger S. 2017. HIF-1α is a key regulator in potentiating suppressor activity and limiting the microbicidal capacity of MDSC-like cells during visceral leishmaniasis. PLoS Pathog 13:e1006616. doi:10.1371/journal.ppat.1006616 28892492 PMC5608422

[59] Roy S, Roy S, Halder S, Jana K, Ukil A. 2024. Leishmania exploits host cAMP/EPAC/calcineurin signaling to induce an IL-33-mediated anti-inflammatory environment for the establishment of infection. J Biol Chem 300:107366. doi:10.1016/j.jbc.2024.107366 38750790 PMC11208913

[60] Vuillefroy de Silly R, Ducimetière L, Yacoub Maroun C, Dietrich P-Y, Derouazi M, Walker PR. 2015. Phenotypic switch of CD8^+^ T cells reactivated under hypoxia toward IL-10 secreting, poorly proliferative effector cells. Eur J Immunol 45:2263–2275. doi:10.1002/eji.201445284 25929785 PMC7163737

[61] Gaber T, Häupl T, Sandig G, Tykwinska K, Fangradt M, Tschirschmann M, Hahne M, Dziurla R, Erekul K, Lautenbach M, Kolar P, Burmester G-R, Buttgereit F. 2009. Adaptation of human CD4^+^ T cells to pathophysiological hypoxia: a transcriptome analysis. J Rheumatol 36:2655–2669. doi:10.3899/jrheum.090255 19884271

[62] Westendorf AM, Skibbe K, Adamczyk A, Buer J, Geffers R, Hansen W, Pastille E, Jendrossek V. 2017. Hypoxia enhances immunosuppression by inhibiting CD4^+^ effector T cell function and promoting treg activity. Cell Physiol Biochem 41:1271–1284. doi:10.1159/000464429 28278498

[63] Ben-Shoshan J, Maysel-Auslender S, Mor A, Keren G, George J. 2008. Hypoxia controls CD4^+^CD25^+^ regulatory T-cell homeostasis via hypoxia-inducible factor-1α. Eur J Immunol 38:2412–2418. doi:10.1002/eji.200838318 18792019

[64] Cho SH, Raybuck AL, Blagih J, Kemboi E, Haase VH, Jones RG, Boothby MR. 2019. Hypoxia-inducible factors in CD4^+^ T cells promote metabolism, switch cytokine secretion, and T cell help in humoral immunity. Proc Natl Acad Sci USA 116:8975–8984. doi:10.1073/pnas.1811702116 30988188 PMC6500120

[65] Cho SH, Raybuck A, Kemboi E, Haase V, Boothby MR. 2019. Hypoxia-Inducible Factors (HIF) in CD4^+^ T cells promote metabolism, switch cytokine secretion, and T cell help in humoral immunity. The Journal of Immunology 202:186. doi:10.4049/jimmunol.202.Supp.186.17 PMC6500120 30988188

[66] Bollinger T, Gies S, Naujoks J, Feldhoff L, Bollinger A, Solbach W, Rupp J. 2014. HIF-1α- and hypoxia-dependent immune responses in human CD4^+^CD25^high^ T cells and T helper 17 cells. J Leukoc Biol 96:305–312. doi:10.1189/jlb.3A0813-426RR 24664971

[67] Roman J, Rangasamy T, Guo J, Sugunan S, Meednu N, Packirisamy G, Shimoda LA, Golding A, Semenza G, Georas SN. 2010. T-cell activation under hypoxic conditions enhances IFN-γ secretion. Am J Respir Cell Mol Biol 42:123–128. doi:10.1165/rcmb.2008-0139OC 19372249 PMC2809218

[68] Wu J, Cui H, Zhu Z, Wang L, Li H, Wang D. 2014. Effect of HIF1α on Foxp3 expression in CD4^+^ CD25^-^ T lymphocytes. Microbiol Immunol 58:409–415. doi:10.1111/1348-0421.12168 24931519

[69] Vignali PDA, DePeaux K, Watson MJ, Ye C, Ford BR, Lontos K, McGaa NK, Scharping NE, Menk AV, Robson SC, Poholek AC, Rivadeneira DB, Delgoffe GM. 2023. Hypoxia drives CD39-dependent suppressor function in exhausted T cells to limit antitumor immunity. Nat Immunol 24:267–279. doi:10.1038/s41590-022-01379-9 36543958 PMC10402660

[70] Chen P-M, Wilson PC, Shyer JA, Veselits M, Steach HR, Cui C, Moeckel G, Clark MR, Craft J. 2020. Kidney tissue hypoxia dictates T cell-mediated injury in murine lupus nephritis. Sci Transl Med 12:eaay1620. doi:10.1126/scitranslmed.aay1620 32269165 PMC8055156

[71] Zenk SF, Vollmer M, Schercher E, Kallert S, Kubis J, Stenger S. 2016. Hypoxia promotes Mycobacterium tuberculosis-specific up-regulation of granulysin in human T cells. Med Microbiol Immunol 205:219–229. doi:10.1007/s00430-015-0442-x 26613797

[72] Volchenkov R, Nygaard V, Sener Z, Skålhegg BS. 2017. Th17 polarization under hypoxia results in increased IL-10 production in a pathogen-independent manner. Front Immunol 8:698. doi:10.3389/fimmu.2017.00698 28674533 PMC5474482

[73] Walter Jackson Iii, Yang Y, Salman S, Dordai D, Lyu Y, Datan E, Drehmer D, Huang T-T, Hwang Y, Semenza GL, Walter Jackson I. 2024. Pharmacologic HIF stabilization activates costimulatory receptor expression to increase antitumor efficacy of adoptive T cell therapy. Sci Adv 10:eadq2366. doi:10.1126/sciadv.adq2366 39196939 PMC11817631

[74] Bannoud N, Dalotto-Moreno T, Kindgard L, García PA, Blidner AG, Mariño KV, Rabinovich GA, Croci DO. 2021. Hypoxia supports differentiation of terminally exhausted CD8 T cells. Front Immunol 12:660944. doi:10.3389/fimmu.2021.660944 34025660 PMC8137905

[75] Finlay DK, Rosenzweig E, Sinclair LV, Feijoo-Carnero C, Hukelmann JL, Rolf J, Panteleyev AA, Okkenhaug K, Cantrell DA. 2012. PDK1 regulation of mTOR and hypoxia-inducible factor 1 integrate metabolism and migration of CD8^+^ T cells. J Exp Med 209:2441–2453. doi:10.1084/jem.20112607 23183047 PMC3526360

[76] Doedens AL, Phan AT, Stradner MH, Fujimoto JK, Nguyen JV, Yang E, Johnson RS, Goldrath AW. 2013. Hypoxia-inducible factors enhance the effector responses of CD8^+^ T cells to persistent antigen. Nat Immunol 14:1173–1182. doi:10.1038/ni.2714 24076634 PMC3977965

[77] Liikanen I, Lauhan C, Quon S, Omilusik K, Phan AT, Bartrolí LB, Ferry A, Goulding J, Chen J, Scott-Browne JP, Yustein JT, Scharping NE, Witherden DA, Goldrath AW. 2021. Hypoxia-inducible factor activity promotes antitumor effector function and tissue residency by CD8^+^ T cells. J Clin Invest 131:e143729. doi:10.1172/JCI143729 33792560 PMC8011896

[78] Novais FO, Scott P. 2015. CD8^+^ T cells in cutaneous leishmaniasis: the good, the bad, and the ugly. Semin Immunopathol 37:251–259. doi:10.1007/s00281-015-0475-7 25800274 PMC4439344

[79] Amorim CF, Lovins V, Singh TP, Novais FO, Harris J, Carvalho LP, Carvalho EM, Beiting DP, Grice EA, Scott P. 2023. Staphylococcus aureus promotes increase IL-1 mediated immunopathology and delayed healing in cutaneous leishmaniasis. J Immun 210:81. doi:10.4049/jimmunol.210.Supp.81.03

[80] Sacramento LA, Farias Amorim C, Campos TM, Saldanha M, Arruda S, Carvalho LP, Beiting DP, Carvalho EM, Novais FO, Scott P. 2023. NKG2D promotes CD8 T cell-mediated cytotoxicity and is associated with treatment failure in human cutaneous leishmaniasis. PLoS Negl Trop Dis 17:e0011552. doi:10.1371/journal.pntd.0011552 37603573 PMC10470908

[81] Farias Amorim C, Lovins VM, Singh TP, Novais FO, Harris JC, Lago AS, Carvalho LP, Carvalho EM, Beiting DP, Scott P, Grice EA. 2023. Multiomic profiling of cutaneous leishmaniasis infections reveals microbiota-driven mechanisms underlying disease severity. Sci Transl Med 15:eadh1469. doi:10.1126/scitranslmed.adh1469 37851822 PMC10627035

[82] Novais FO, Carvalho LP, Graff JW, Beiting DP, Ruthel G, Roos DS, Betts MR, Goldschmidt MH, Wilson ME, de Oliveira CI, Scott P. 2013. Cytotoxic T cells mediate pathology and metastasis in cutaneous leishmaniasis. PLoS Pathog 9:e1003504. doi:10.1371/journal.ppat.1003504 23874205 PMC3715507

[83] Amorim CF, Novais FO, Nguyen BT, Misic AM, Carvalho LP, Carvalho EM, Beiting DP, Scott P. 2019. Variable gene expression and parasite load predict treatment outcome in cutaneous leishmaniasis. Sci Transl Med 11:eaax4204. doi:10.1126/scitranslmed.aax4204 31748229 PMC7068779

[84] Novais FO, Nguyen BT, Scott P. 2021. Granzyme B inhibition by tofacitinib blocks the pathology induced by CD8 T cells in cutaneous leishmaniasis. J Invest Dermatol 141:575–585. doi:10.1016/j.jid.2020.07.011 32738245 PMC7855313

[85] Novais FO, Carvalho LP, Passos S, Roos DS, Carvalho EM, Scott P, Beiting DP. 2015. Genomic profiling of human Leishmania braziliensis lesions identifies transcriptional modules associated with cutaneous immunopathology. J Invest Dermatol 135:94–101. doi:10.1038/jid.2014.305 25036052 PMC4268311

[86] Crosby EJ, Clark M, Novais FO, Wherry EJ, Scott P. 2015. Lymphocytic choriomeningitis virus expands a population of NKG2D^+^CD8^+^ T cells that exacerbates disease in mice coinfected with Leishmania major. J Immunol 195:3301–3310. doi:10.4049/jimmunol.1500855 26290604 PMC4575880

[87] Amorim Sacramento L, Farias Amorim C, G Lombana C, Beiting D, Novais F, P Carvalho L, M Carvalho E, Scott P. 2024. CCR5 promotes the migration of pathological CD8^+^ T cells to the leishmanial lesions. PLoS Pathog 20:e1012211. doi:10.1371/journal.ppat.1012211 38709823 PMC11098486

[88] Novais FO, Carvalho AM, Clark ML, Carvalho LP, Beiting DP, Brodsky IE, Carvalho EM, Scott P. 2017. CD8^+^ T cell cytotoxicity mediates pathology in the skin by inflammasome activation and IL-1β production. PLoS Pathog 13:e1006196. doi:10.1371/journal.ppat.1006196 28192528 PMC5325592

[89] Larsen SB, Cowley CJ, Fuchs E. 2020. Epithelial cells: liaisons of immunity. Curr Opin Immunol 62:45–53. doi:10.1016/j.coi.2019.11.004 31874430 PMC7067656

[90] Paduch K, Debus A, Rai B, Schleicher U, Bogdan C. 2019. Resolution of cutaneous leishmaniasis and persistence of Leishmania major in the absence of arginase 1. J Immunol 202:1453–1464. doi:10.4049/jimmunol.1801249 30665936

[91] Boutin AT, Weidemann A, Fu Z, Mesropian L, Gradin K, Jamora C, Wiesener M, Eckardt K-U, Koch CJ, Ellies LG, Haddad G, Haase VH, Simon MC, Poellinger L, Powell FL, Johnson RS. 2008. Epidermal sensing of oxygen is essential for systemic hypoxic response. Cell 133:223–234. doi:10.1016/j.cell.2008.02.038 18423195 PMC2849644

[92] Peyssonnaux C, Boutin AT, Zinkernagel AS, Datta V, Nizet V, Johnson RS. 2008. Critical role of HIF-1α in keratinocyte defense against bacterial infection. J Invest Dermatol 128:1964–1968. doi:10.1038/jid.2008.27 18323789

[93] Konieczny P, Xing Y, Sidhu I, Subudhi I, Mansfield KP, Hsieh B, Biancur DE, Larsen SB, Cammer M, Li D, Landén NX, Loomis C, Heguy A, Tikhonova AN, Tsirigos A, Naik S. 2022. Interleukin-17 governs hypoxic adaptation of injured epithelium. Science 377:eabg9302. doi:10.1126/science.abg9302 35709248 PMC9753231

[94] Sen Santara S, Roy J, Mukherjee S, Bose M, Saha R, Adak S. 2013. Globin-coupled heme containing oxygen sensor soluble adenylate cyclase in Leishmania prevents cell death during hypoxia. Proc Natl Acad Sci USA 110:16790–16795. doi:10.1073/pnas.1304145110 24082109 PMC3801027

[95] Ayres DC, Pinto LA, Giorgio S. 2008. Efficacy of pentavalent antimony, amphotericin B, and miltefosine in Leishmania amazonensis-infected macrophages under normoxic and hypoxic conditions. J Parasitol 94:1415–1417. doi:10.1645/GE-1613.1 18576874

[96] Murray HW, Xiang Z, Ma X. 2006. Responses to Leishmania donovani in mice deficient in both phagocyte oxidase and inducible nitric oxide synthase. Am J Trop Med Hyg 74:1013–1015. doi:10.4269/ajtmh.2006.74.1013 16760512

